# Effects of Ladder-Climbing Exercise on Mammary Cancer: Data from a Chemically Induced Rat Model

**DOI:** 10.3390/vetsci12040303

**Published:** 2025-03-26

**Authors:** Jessica Silva, Tiago Azevedo, Inês Aires, Catarina Medeiros, Maria J. Neuparth, Fernanda Seixas, Rita Ferreira, Ana I. Faustino-Rocha, Paula A. Oliveira, José Alberto Duarte

**Affiliations:** 1 Centre for the Research and Technology of Agro-Environmental and Biological Sciences (CITAB), University of Trás-os-Montes and Alto Douro (UTAD), 5000-801 Vila Real, Portugal; silva_jessy@hotmail.com (J.S.); tiagoaazevedo99@gmail.com (T.A.); iaa@ua.pt (I.A.); catarinamedeiros2508@outlook.com (C.M.); pamo@utad.pt (P.A.O.); 2 Institute for Innovation, Capacity Building and Sustainability of Agri-Food Production (Inov4Agro), University of Trás-os-Montes and Alto Douro (UTAD), 5000-801 Vila Real, Portugal; 3 Animal and Veterinary Research Centre (CECAV), Associate Laboratory for Animal and Veterinary Science—AL4AnimalS, University of Trás-os-Montes and Alto Douro (UTAD), 5000-801 Vila Real, Portugal; fseixas@utad.pt; 4 Mountain Research Center (CIMO), Associated Laboratory for Sustainability and Technology in Inland Regions (SusTEC), Polytechnique Institute of Bragança (IPB), 5300-253 Bragança, Portugal; 5 LAQV-REQUIMTE, Department of Chemistry, University of Aveiro, 3810-193 Aveiro, Portugal; ritaferreira@ua.pt; 6 Research Center in Physical Activity, Health and Leisure (CIAFEL), Faculty of Sports, University of Porto (FADEUP), 4200-450 Porto, Portugal; Laboratory for Integrative and Translational Research in Population Health (ITR), 4200-450 Porto, Portugal; Toxicology Research Unit (TOXRUN), University Institute of Health Sciences—CESPU, 4585-116 Gandra, Portugal; mneuparth@hotmail.com; 7 Department of Zootechnics, School of Sciences and Technology, University of Évora, 7004-516 Évora, Portugal; 8 Comprehensive Health Research Center (CHRC), University of Évora, 7004-516 Évora, Portugal; 9 Department of Veterinary Sciences, University of Trás-os-Montes and Alto Douro (UTAD), 5000-801 Vila Real, Portugal; 10 Associate Laboratory i4HB, Institute for Health and Bioeconomy, University Institute of Health Sciences—CESPU, 4585-116 Gandra, Portugal; jose.duarte@iucs.cespu.pt; 11 UCIBIO—Applied Molecular Biosciences Unit, Translational Toxicology Research Laboratory, University Institute of Health Sciences (1H-TOXRUN, IUCS-CESPU), 4585-116 Gandra, Portugal

**Keywords:** breast cancer, physical activity, resistance training, Wistar rats

## Abstract

Breast cancer remains a significant global health issue, affecting both humans and companion animals, particularly female dogs and cats, where mammary tumors are among the most common cancers. Strategies to minimize the impact of this disease on patients, pet owners, and veterinary medicine are essential. This study analyses the effects of resistance training on the development of chemically induced mammary cancer in female Wistar rats, divided into four groups: sedentary control (CTR), sedentary induced (CTR+*N*-methyl-*N*-nitrosourea (MNU)), exercised control (EX), and exercised induced (EX+MNU). The exercise protocol involved ladder climbing three times a week for 18 weeks with the load progressively increasing. At the study’s end, blood and histopathological samples were collected and analyzed. Although tumor onset occurred two weeks earlier and incidence was slightly higher in the exercised group (EX+MNU) compared to the control group (CTR+MNU), the mortality rate was lower, and the malignancy was not as aggressive. No systemic inflammation was observed, as the levels of albumin, C-reactive protein (CRP), and interleukin 6 (IL-6) in the MNU groups remained similar to the controls. Exercise has been shown to promote overall health by increasing physical fitness, boosting immunological function, and improving metabolic health. These findings may offer valuable insights into the potential role of resistance training in managing mammary cancer in companion animals. However, further research is required to assess clinical applicability and to establish safe and effective exercise protocols for veterinary oncology.

## Simple Summary

Breast cancer is a major health concern for both humans and companion animals, particularly female dogs and cats. This study explores whether resistance training can influence the development of mammary cancer in female rats. The rats were divided into four groups, including those that exercised by climbing a ladder with increasing weight three times a week for 18 weeks. At the end of the study, tumor development, inflammation, and overall health were assessed. While tumors appeared slightly earlier and in more animals that exercised, these tumors were less aggressive, and the mortality rate was lower. Importantly, no signs of harmful systemic inflammation were found. Exercise is known to support health by improving fitness, strengthening immunity, and enhancing metabolism. These findings suggest that resistance training may have potential benefits in managing mammary cancer in pets. However, more research is needed to confirm how exercise can be safely and effectively used in veterinary medicine.

## 1. Introduction

Breast cancer is a significant health concern not only in humans but also in companion animals, particularly in female dogs and cats, where mammary tumors are among the most common cancers diagnosed [1]. While early detection and advanced treatments have improved survival rates, there is a growing need for effective preventive strategies [2,3,4]. In veterinary medicine, non-pharmacological interventions, such as exercise training, have gained attention for their potential role in cancer prevention and management [5,6].

The current literature provides strong evidence that resistance training offers numerous health benefits. This type of exercise has been shown to combat the loss of muscle mass and strength caused by aging, illness, or prolonged inactivity [7,8]. It can also aid in the recovery from muscle and tendons injuries by strengthening tissues, improving flexibility, and enhancing circulation to the affected areas [9,10]. Additionally, resistance training helps manage obesity by increasing metabolic rate promoting fat loss and improving body composition through the growth of lean muscle tissue [11]. The potential of resistance exercise as a safe and effective adjuvant for cancer treatment is an increasingly popular area of study [12]. The purpose of this study was to investigate the effects of resistance training on the development of chemically induced mammary cancer in a rat model.

Laboratory animals have been essential in advancing breast cancer research, offering crucial insights into the disease’s biological mechanisms and paving the way for promising preventive and therapeutic approaches [13]. Among these animals, rats occupy a prominent position in mammary cancer research [14,15]. Their advantageous use to study breast cancer can be attributed to several factors, including genetic similarity to humans and other mammals, a well-characterized mammary gland, and the development of mammary tumors histologically similar with those observed in women [13,16,17].

Ladder climbing is a form of resistance exercise that engages multiple muscle groups in both the upper and lower limbs and has been shown to produce beneficial physiological effects in animals [18]. This resistance training has led to improvements in metabolism, immune function, and hormonal balance [19,20]. These changes are believed to contribute to the reduced risk of cancer by potentially slowing the growth of cancer cells or making the body more efficient at detecting and destroying them [21,22]. Ladder training positively impacts both muscle strength and endurance through a combination of increased muscle size, improved neuromuscular coordination, and optimized metabolic function [23]. As training intensity increases, a greater number of muscle fibers are recruited, particularly those responsible for rapid, powerful movements [24]. Additionally, metabolic adaptations enhance the muscle efficiency in oxygen use, enhancing endurance and delayed fatigue. Resistance training also strengthens the communication between the nervous system and muscles, increasing both strength and movement efficiency [23]. Given the limited research on the effects of ladder-climbing exercise on mammary cancer in companion animals, this study aims to investigate the influence of resistance training on breast cancer prevention and the progression of chemically induced mammary tumors in a rat model. Findings from this study could provide valuable insights for veterinary medicine and the development of exercise-based interventions for pets at risk of developing mammary cancer.

## 2. Materials and Methods

### 2.1. Ethical Approval

The study was conducted in accordance with the biosecurity standards for animal models (European Directive 2010/60/EU and National Decree-Law 113/2013). The experimental protocol was approved by the Portuguese Ethics Committee for Animal Experimentation (Direção Geral de Alimentação e Veterinária; approval No. 04583) and underwent review by an Ethics Review Body (Órgão Responsável pelo Bem-Estar dos Animais, ref.834-e-CITAB-2020).

### 2.2. Animals and Experimental Design

**Figure 1:**
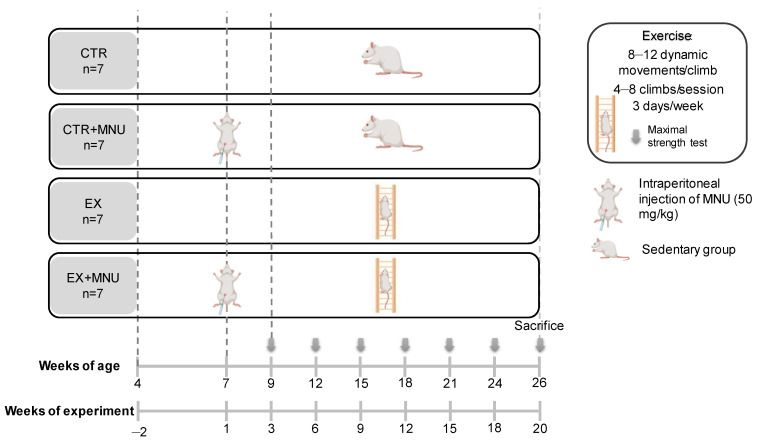
Experimental design. CTR: sedentary control group; CTR+MNU: sedentary induced group; EX: exercised control group and EX+MNU: exercised induced group.

Twenty-eight 4-week-old female Wistar rats (Rattus norvegicus) were purchased from Envigo RMS Spain S.L. (Barcelona, Spain) and were housed at the animal facilities of the University of Trás-os-Montes and Alto Douro. Environmental conditions, including temperature (23 ± 2 °C), humidity (50 ± 10%), and a 12:12 h light (8 a.m.): dark (8 p.m.) cycle, were strictly controlled, and tap water and food (2014 Teklad Global Rodent diet, Envigo, Spain) were provided ad libitum. The animals were randomly assigned to one of four groups (n = 7 per group): sedentary control (CTR), sedentary induced (CTR+*N*-methyl-*N*-nitrosourea (MNU)), exercised control (EX), and exercised induced (EX+MNU) (Figure 1). On the 50th day of age, animals from the MNU-induced groups received an intraperitoneal injection of the carcinogen MNU (50 mg/kg of body weight) (Fluorochem, UK) dissolved in 0.9% saline solution (NaCl 0.9%, B. Braun, Germany). A vehicle of 0.5 mL NaCl 0.9% (B. Braun, Germany) was intraperitoneally administered to both non-induced groups. Seven days after the MNU injection, the health status of the animals was assessed once a week, following the recommendations of a previously published table of humane endpoints. This table evaluates the following parameters: general appearance, behavior, and clinical signs such as hydration status evaluated by gently lifting the skin on the animals’ backs, noticing that the skin does not immediately snap back due to diminished turgor, and body temperature, measured with a thermometer [25]. Palpation of the mammary chains was conducted twice a week by two investigators. The time of the first tumor appearance and the overall number of tumors were documented throughout the experiment. The animals’ body weight was measured every week (KERN^®^ PLT 6200-2A, Dias de Sousa S.A., Alcochete, Portugal), and accurate body weight was determined by subtracting the tumor weight from the final body weight [26].

### 2.3. Exercise Training

Animals were trained three days a week for 18 consecutive weeks, starting two weeks after the MNU injection, with the intent to evaluate its preventive effects (Figure 1). All training sessions were conducted under low light conditions to minimize stress.

The exercise training consisted of a homemade ladder climbing with dimensions of 1.10 m in height and 0.2 m in width, featuring a 4 mm grid an inclination of 80 degrees. A dark-covered chamber was built at the top of the ladder to allow for interval resting between climbs (1 min). To minimize the stress levels associated with using the apparatus, the animals were familiarized with it by voluntarily climbing it once a day for five days during the week before the commencement of the exercise protocol. The rats were positioned at the lower section of the ladder and encouraged to climb by gentle manual prompting them to initiate movement until successfully completed a full climb. During this period, no additional load was applied. The exercise protocol was adapted from the one described by Padilha et al. [27]. During each session, exercised animals completed 4–8 climbs, with 8–12 dynamic movements for each climb, carrying 80% of their maximal carrying load (MCL).

### 2.4. Maximal Strength Test

Prior to the beginning of the exercise training protocol, a maximal strength test was conducted in accordance with the methodology previously described by Padilha et al. [27]. This test was performed every three weeks, including the final session of exercise (see Figure 1). All animals started climbing with load equivalent to 75% of their own body weight attached to their tail. After a successful climb, an additional 30 g load was attached to the tail. This process was repeated until the animal was unable to complete the full climb for three consecutive attempts. In such an instance, the weight load pulled during the last successful climb was considered the animal’s MCL.

### 2.5. Sacrifice and Necropsy of the Animals

At the end of the experiment, all animals were subjected to a 12-h fasting period. Naso-anal length was measured in order to calculate body mass index (final body weight/naso-anal length squared) [28,29]. Before the animals were anesthetized, the abdominal and dorsal regions were shaved, taking care not to damage the nipples, in order to remove the animals’ skin as previously described by our team [30]. The animals were then euthanized via an intraperitoneal injection of ketamine (75 mg/kg, Imalgene 1000, Merial SA, Lyon, France) and xylazine (10 mg/kg, Rompun 20%, Bayer Healthcare S.A., Kiel, Germany), followed by exsanguination via cardiac puncture [31]. Blood samples were collected directly from the heart and placed in lithium-heparin and EDTA tubes; then, the lithium-heparin tubes were centrifuged (Heraeus Labofuge 400R, Thermo Fisher Scientific, Waltham, MA, USA) at 1500× *g* for 15 min. The resulting plasma was stored at −80 °C for subsequent biochemical analyses. EDTA tubes were used for hematological analysis. Each animal was examined and inspected for the presence of mammary tumors; for this purpose, the skin was removed and carefully examined under light to detect small mammary tumors not previously identified by palpation, as previously described by our team [30]. Subsequently, the weights of the mammary tumors and the abdominal relative organs (spleen, heart, lungs, liver, soleus, gastrocnemius, and biceps brachii) were calculated as the ratio of the organs weight (g) to the animal’s body weight (g), and the relative length of the bones was calculated as the ratio of the bones length (cm) to the animals naso-anal length (cm). The samples were preserved in 10% phosphate-buffered formaldehyde for a minimum of 24 h.

### 2.6. Blood Samples Analysis

A complete blood count (CBC) was done using the IDEXX ProCyte Dx Hematology system (IDEXX Laboratories, Inc., Westbrook, ME, USA). The CBC included several parameters, such as erythrocytes, hematocrit, hemoglobin, red cells distribution width (RDW), reticulocytes, leukocytes, neutrophils, lymphocytes, monocytes, eosinophils, basophils, neutrophil–lymphocyte ratio (NLR), platelets, mean platelet volume (MPV), and platelet distribution width (PDW).

Biochemical parameters, including albumin, cholesterol, creatinine kinase-MB (CK-MB), and triglycerides, were analyzed using the Pertige 24i autoanalyzer (Pertige 24i, Cormay S.A., PZ, Łomianki, Poland).

Serum samples were diluted 1:20 in Tris-buffered saline (TBS; 10 mM Tris, pH 8.0, 0.15 M NaCl). Then, 50 µL of each diluted sample was blotted using a vacuum onto a nitrocellulose membrane (Amersham™, Protan^®^, GE Healthcare, Chicago, IL, USA), previously activated with 10% methanol solution. Following this, membranes were stained with Ponceau S to control protein loading. Nonspecific binding was blocked with 5% (*w*/*v*) non-fat dry milk in TBS-T (TBS with 0.5% Tween 20) for 90 min at room temperature with agitation. The membranes were then incubated with the corresponding primary antibodies (Myostatin (rabbit, ab98337, Abcam, UK), C-reactive protein (CRP) (rabbit, ab65842, Abcam, UK), interleukin 6 (IL-6; rabbit, ab6672, Abcam, UK), and vascular endothelial growth factor-A (VEGF-A; rabbit, ab46154, Abcam, UK) (diluted 1:1000 in 5% (*w*/*v*) non-fat dry milk in TBS-T)) overnight, at 4 °C with shaking. After, membranes were washed three times with TBS-T for 10 min each and then incubated with the specific secondary antibody (anti-rabbit, NA934V, GE Healthcare) (diluted 1:1000 in 5% (*w*/*v*) non-fat dry milk in TBS-T) for 90 min at room temperature. Lastly, membranes were washed again three times with TBS-T. The Immunoreactive bands were detected using ECL reagent (WesternBright™ ECL Advansta, San Jose, CA, USA) according to the manufacturer’s procedure. Images were acquired using the ChemiDoc XR System (Bio-Rad^®^ Hercules, CA, USA) and analyzed with Image Lab software (Bio-Rad^®^, CA, USA, version 6.0.0). The optical densities obtained were expressed in arbitrary units.

### 2.7. Histological and Immunohistochemical Analysis of Mammary Tumors

Following the sacrifice of the animals, all mammary tumors underwent standard histological processing. The tissues were fixed in 10% buffered formalin, embedded in paraffin, and three µm sections were stained with hematoxylin and eosin (H&E). An experienced pathologist examined mammary tumors under a light microscope, using the Russo and Russo criteria [32].

The Novolink Polymer Detection System (Leica Biosystems, Newcastle, UK) was used for the immunohistochemical detection of estrogen receptor alpha (ERα), progesterone receptor (PR), Ki-67, CD8, and CD163 in mammary tumors. The primary antibodies used were anti-ERα (clone 6F11, 1:500 dilution, Novocastra, Newcastle, UK), anti-PR (ab16661, 1:300, Abcam, Cambridge, UK), anti-Ki-67 antigen (ab16667, 1:200, Abcam, The Netherlands), anti-CD8 (ab33786, Abcam, Cambridge, UK), and anti-CD163 (ab182422, 1:500, Abcam, Cambridge, UK). The sections were incubated overnight at 4 °C. To quantify the immunoexpression of ERα, PR, and Ki-67, a minimum of 1000 neoplastic cells per mammary tumor were evaluated. The images captured with a 40× objective of an Eclipse E600 microscope (Nikon, Tokyo, Japan) were examined using the ImmunoRatio 1.0 plugin in the ImageJ program. The data are presented as a percentage of neoplastic cells that exhibited immunopositivity. CD8 (intra- and extra-tumoral staining) and CD163 immunoexpression were quantified by counting the number of positive cells per area (number of positive cells per mm^2^) in the image captured with a 40× objective (Eclipse E600 microscope, Nikon, Tokyo, Japan).

A histochemical analysis with a double staining was conducted using toluidine blue and fuchsin orange G to determine the density of mast cells. The mean number of mast cells was determined following the analysis of 10 amplified fields at 400× magnification [33].

### 2.8. Statistical Analysis

The data were statistically analyzed using SPSS version 26 (Chicago, IL, USA). The comparison of continuous variables among groups was conducted using two-way ANOVA, with the application of Tukey’s multiple comparison test. The Chi-square test was used to determine the relationship between the number of tumors, histopathological tumors, and groups. Differences between the groups in terms of maximal carrying load and immunohistochemical evaluation were analyzed using the independent samples *t*-test. The results are presented as mean ± standard deviation (S.D.), with statistical significance set at *p* < 0.05.

## 3. Results

### 3.1. General Findings

The animals in the exercised groups were successfully acclimated to the exercise routine. Two animals from the CTR+MNU group and one from the EX+MNU group were sacrificed due to changes in humane endpoint parameters, such as general appearance (decreased body weight and lack of grooming), alterations in behavior (including unresponsiveness to external stimuli), and clinical sings such as dehydration. They were sacrificed in the 12th and 17th weeks due to a deterioration in their health conditions, resulting in mortality rates of 28.6% and 14.3% in CTR+MNU and EX+MNU groups. These animals were excluded from all subsequent analyses.

We observed that the MNU-induced groups (CTR+MNU and EX+MNU) exhibited a lower final body weight compared to the CTR and EX groups. However, these changes did not reach statistical significance (*p* > 0.05; Table 1). No differences were found in initial body weight and in body mass index among groups (*p* > 0.05).

### 3.2. Maximal Strength Test

**Figure 2:**
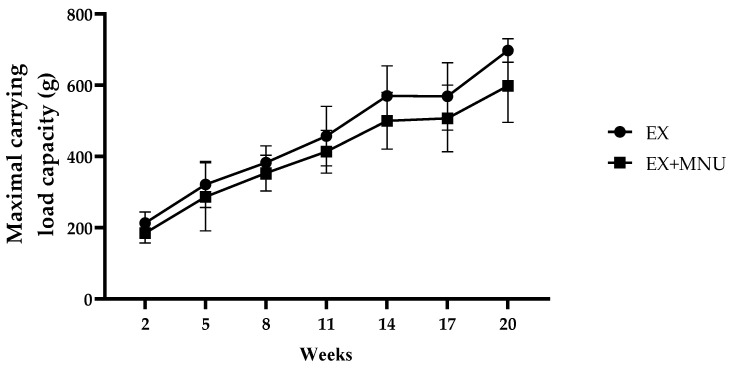
Maximal carrying load capacity (MCL) per maximal strength test in both exercised groups (EX and EX+MNU) (mean ± S.D.). EX: exercised control group, and EX+MNU: exercised induced group.

The MCL (g) per training session was evaluated over the course of 18 weeks, encompassing a total of 47 training sessions. A gradual increase in the MCL was observed in both groups over the experiment. It was also found that the animals from the induced group (EX+MNU group) consistently exhibited a lower load capacity in comparison to those who underwent exercise alone (EX group). By the final week of the study, a statistically significant difference was observed between the two groups, with the EX group displaying a higher MCL in comparison to the EX+MNU group (*p* < 0.05; Figure 2).

No significant alterations were found in the relative weights of the spleen, heart, lungs, liver, soleus, gastrocnemius, and biceps brachii muscles, as well as the length of the femur among the experimental groups (*p* > 0.05; Table 2). However, a significant difference was observed in relative lengths of the tibias between the EX and CTR groups (*p* < 0.05).

### 3.3. Blood Samples Analysis

The results of the analysis of blood samples are displayed in Table 3 and Table 4. An elevation in erythrocyte, hemoglobin, and hematocrit levels was observed in the EX+MNU group when compared to the EX and CTR+MNU groups (*p* < 0.05). A tendency towards a reduction in reticulocytes was observed in the EX group compared to the CTR and EX+MNU groups. The mean leukocyte count was found to be lower in the CTR and EX groups compared to the CTR+MNU and EX+MNU groups, respectively (*p* < 0.05), with the high number of lymphocytes contributing to this difference.

No significant changes were observed in albumin, cholesterol, and triglycerides. However, the creatinine kinase-MB showed a tendency, in the exercised groups (EX and EX+MNU), to have lower values (*p* > 0.05; Table 4).

**Figure 3:**
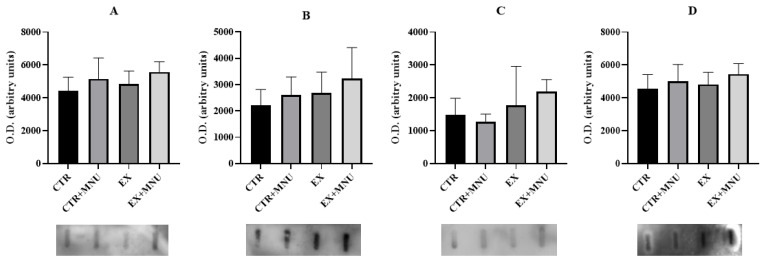
Circulating levels of myostatin (**A**), C-reactive protein (CRP) (**B**), interleukin-6 (IL-6) (**C**), and vascular endothelial growth factor-A (VEGF-A) (**D**). Values are presented as mean ± S.D. in each group in optical density (OD). CTR: sedentary control group; CTR+MNU: sedentary induced group; EX: exercised control group and EX+MNU: exercised induced group. Statistically significant differences were not found (*p* > 0.05).

No statistically significant differences were observed in the circulating levels of myostatin, CRP, IL-6, and VEGF-A (*p* > 0.05; Figure 3).

### 3.4. Mammary Tumours

**Figure 4:**
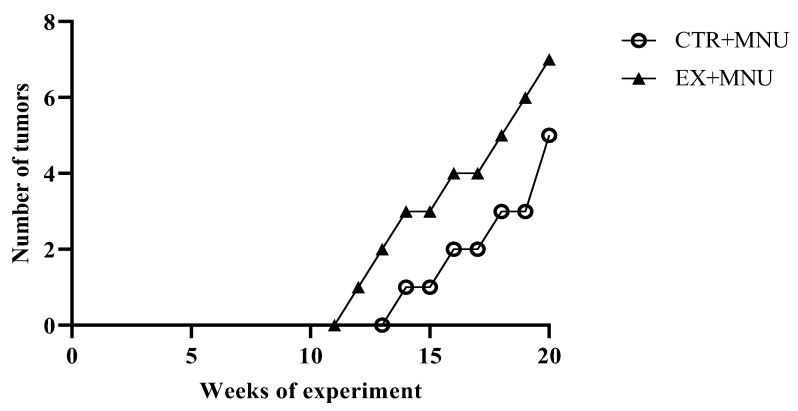
Effects of resistance training on the carcinogenic response of the mammary gland: cumulative number of mammary tumors in both MNU-induced groups (CTR+MNU and EX+MNU) throughout the experiment. CTR+MNU: sedentary induced group, and EX+MNU: exercised induced group.

Animals in the CTR and EX groups did not develop mammary tumors. The latency period, i.e., the time from carcinogen administration to the appearance of the first tumor, was shorter in the EX+MNU group (12 weeks), than in CTR+MNU (14 weeks). The incidence of tumors was also slightly higher in the EX+MNU group; four out of six animals developed tumors (incidence of 67%). The CTR+MNU group showed an incidence of 60% (three out of five animals developed tumors). At the end of the experiment, the CTR+MNU group exhibited a lower tumor burden than the EX+MNU group, with five tumors versus seven tumors, respectively (Figure 4).

### 3.5. Histopathological, Histochemical and Immunohistochemistry Analysis

The histopathological characterization of the mammary tumors is shown in Table 5. The animals from CTR+MNU group developed five malignant invasive carcinomas. The animals from EX+MNU group developed seven malignant carcinomas, from which one was an in situ carcinoma and the remaining six were invasive.

**Figure 5:**
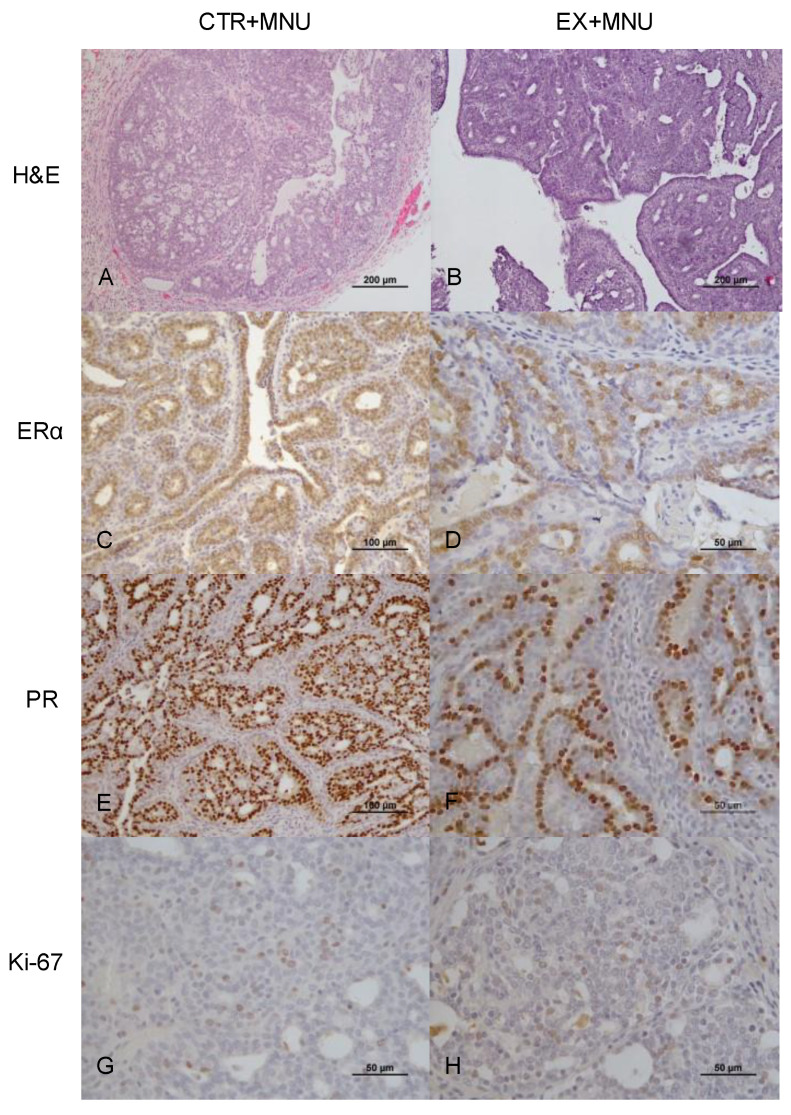
H&E staining (**A**,**B**), and immunohistochemical staining for ERα (**C**,**D**), PR (**E**,**F**), and Ki-67 (**G**,**H**) in mammary tumors from the CTR+MNU (**A**,**C**,**E**,**G**) and EX+MNU (**B**,**D**,**F**,**H**) groups. Immunostaining was observed in the nuclei of epithelial cells. Statistically significant differences were found in the immunoexpression of ERα and PR between groups (*p* < 0.05). CTR+MNU: sedentary induced group, and EX+MNU: exercised induced group.

Mammary tumors in both CTR+MNU and EX+MNU groups were immunopositive for ERα (Figure 5C,D), PR (Figure 5E,F), and Ki-67 (Figure 5G,H). Ki-67 immunostaining did not appear to differ between the CTR+MNU and EX+MNU groups (Table 6). The immunoexpression of ERα and progesterone receptor in invasive cribriform carcinomas was significantly lower in the EX+MNU group compared to the CTR+MNU group (*p* = 0.031 and *p* = 0.013, respectively). Regarding Ki-67 immunoexpression, invasive carcinomas in the CTR+MNU group displayed significant differences between papillary and cribriform subtypes (*p* < 0.0001). Similar differences were observed in papillary invasive carcinomas between the CTR+MNU and EX+MNU groups (*p* = 0.002). Additionally, a significant difference in Ki-67 immunoexpression was found between in situ carcinomas and papillary invasive carcinomas in the EX+MNU group (*p* = 0.047).

**Figure 6:**
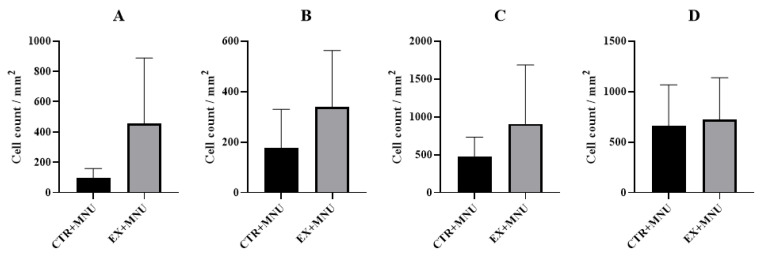
Immunoexpression of CD8 (**A**), intra-tumoral CD163 (**B**), extra-tumoral CD163 (**C**), and histochemical analysis of a double staining with toluidine blue and fuchsin orange G (**D**) in MNU-induced mammary tumors in both CTR+MNU and EX+MNU groups. Values are presented as mean ± S.D. in each group. CTR+MNU: sedentary induced group, and EX+MNU: exercised induced group. Statistically significant differences were not found (*p* > 0.05).

**Figure 7:**
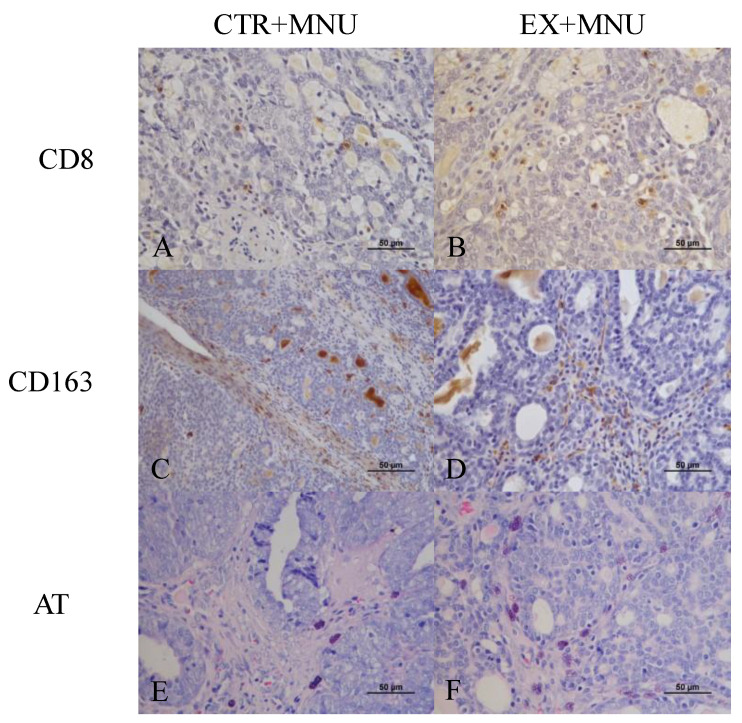
Immunoexpression of CD8 (**A**,**B**), CD163 (**C**,**D**), and double staining with toluidine blue and fuchsin orange G (**E**,**F**) in mammary tumors from the CTR+MNU (**A**,**C**,**E**) and EX+MNU (**B**,**D**,**F**) groups. Immunostaining was observed in the cytoplasm of epithelial and stromal cells (**A**–**D**). secretory granules of mast cells are stained in purple. Statistically significant differences were not found (*p* > 0.05). CTR+MNU: sedentary induced group, and EX+MNU: exercised induced group.

The count of cytotoxic T cells per mm^2^ was evaluated using CD8 immunostaining, revealing a higher count of CD8-positive cells in the EX+MNU group than in the CTR+MNU group (*p* = 0.0699) (Figure 6A and Figure 7A,B). The EX+MNU group exhibited greater positivity per mm^2^ when compared to the CTR+MNU group, with higher intra-tumoral (*p* = 0.0555) than extra-tumoral (*p* = 0.1690) CD163 immunoexpression (Figure 6B,C and Figure 7C,D). The histochemical analysis of double staining with toluidine blue and fuchsin orange G revealed no statistically significant differences in the number of mast cells between CTR+MNU and EX+MNU groups (*p* = 0.8644) (Figure 6D and Figure 7E,F).

## 4. Discussion

In this research, we observed a reduction of mortality rate between the EX+MNU group (14.3%) and the CTR+MNU group (28.6%; *p* > 0.05), suggesting that exercise may have a protective effect, potentially mitigating the harmful impact of MNU in animals’ health. Although no statistically significant differences were observed in the body weight of the animals, the induced groups (CTR+MNU and EX+MNU groups) showed a tendency towards reduced body weight compared to the non-induced groups (EX and CTR groups) by the end of the protocol. This reduction in body weight may be linked to the overall negative impact of MNU on the animals’ health, as reported in similar studies, such as that of Ashrafi et al., which showed lower body weight in MNU-induced animals [34].

To determine the maximum load that each animal can carry, many authors have employed the MCL as a measure. Despite the absence of differences in the relative weights of the muscles (soleus, gastrocnemius, and biceps brachii), a gradual increase in the MCL was observed over time. Souza et al. reported a similar gradual increase in the exercise control group of adult female Holtzman rats [35]. Although the improvement was somewhat diminished in the presence of cancer, it was still evident, underscoring the beneficial effects of exercise on muscle function.

The analysis of the spleen, heart, lungs, and liver did not reveal any differences between groups. Additionally, an increase in relative tibia length was observed in the exercised group (EX) compared to the control group (CTR), suggesting that resistance exercise likely promotes an increase in bone length (at the time of sacrifice, they were the equivalent of 18 years in human age) [36]. The literature presents contradictory findings regarding tibia length; Souza et al. found no differences between the control and exercise groups in adult female Holtzman rats after 12 weeks of resistance exercise [35].

Our study’s findings suggest that mammary cancer has an impact on hematological parameters, because we found an elevated levels of erythrocytes, hematocrit, and hemoglobin observed in the EX+MNU compared to EX group. This finding implies that the presence of mammary cancer may lead to significant changes in blood composition, potentially as a physiological response to the disease or its progression. However, there are no comparable studies in literature that support our results.

It is well known that cancer can lead to systemic inflammation [37], and in our study, we noticed a trend in the control groups (CTR and EX) showing lower leukocytes values compared to the respective induced groups (CTR+MNU and EX+MNU). Conversely, we observed a trend towards a decrease in lymphocytes in the control groups (CTR and EX) compared to the induced groups (CTR+MNU and EX+MNU), which may reflect the tumor-induced immunosuppression and the exhaustion of adaptive cells [38]. Similar trends were found by Emam et al., who noted a decrease in leukocytes and therefore a decrease in neutrophils and lymphocytes in control groups compared to MNU-induced groups in female Sprague–Dawley rats after 20 weeks [39].

Although the hematological results suggest an increase in WBC, which could indicate systemic inflammation, the lack of elevated levels of albumin, CRP, and IL-6 suggests that cancer did not cause systemic inflammation. This discrepancy may indicate that the expected systemic inflammation commonly associated with mammary cancer was not significantly observed in our study [40,41]. Furthermore, the lack of significant differences in VEGF levels between groups could suggest that angiogenesis triggered by mammary tumors was not activated.

The EX+MNU group showed a slightly higher number of tumors (n = 7), compared to the CTR+MNU group (n = 5), with a marginally increased incidence (67% versus 60%). Although the first tumor appeared in the EX+MNU group, they appeared at similar times in both groups, suggesting no noticeable effect of exercise on the onset of tumor development. Asharafi et al. injected female Wistar rats with MNU at the same dose at three different time points (50, 65, and 80 days of age) and achieved only 65% tumor incidence. In contrast, other studies have reported a tumor incidence of 100% in female Sprague–Dawley rats administered with the same carcinogen at 49–56 days of age [42,43,44,45]. However, these results are not meaningful to conclude that exercise had a real impact on increasing tumor incidence. Additionally, the EX+MNU group exhibited a more diverse range tumor histology, with both in situ and invasive tumors, hinting at a potential influence of exercise on tumor type and possibly aggressiveness. However, no significant differences were observed in tumor proliferation (Ki-67), suggesting that exercise had little to no effect on tumor evolution, and the lack of significant results raises the question of why exercise had minimal or no impact in this context.

The lower expression of ERα- and PR-positive cells in the EX+MNU group compared to CTR+MNU group suggests that the tumors may be less dependent on hormonal signaling for growth. This shift could indicate a more aggressive tumor characteristics, as ERα- and PR-negative tumors are typically more aggressive and often harder to treat. Given that resistance exercise may not affect tumors in the same way as other forms of exercise, there are no studies available that demonstrate or validate our findings regarding ERα and PR expression in response to ladder resistance training.

Regarding tumor infiltrate, the increased presence of CD8 cells observed in the EX+MNU group suggests enhanced anti-tumor immunity. CD8 cells are essential immune cells that target and destroy cancer cells; their increased presence in tumors typically leads to a stronger immune response and reduced tumor aggressiveness [46]. Similar results were found by Qin et al., who used female BALB/c mice subjected to resistance training on a ladder and aerobic rotarod exercise for 21 days each [47]. CD163-positive cells, which can have both pro- and anti-tumor activity, increased in the EX+MNU group, indicating changes in immune responses and tissue remodeling, as demonstrated by double immunostaining. Overall, 18 weeks of resistance training appears to influence the expression of hormonal receptors and immune cell infiltration in mammary tumors, potentially leading to less aggressive tumor behavior and an enhanced immune response. To date, no literature specifically supports our findings. The double staining used to determine mast cells density was not affected by exercise training. Mast cell density in the EX+MNU and CTR+MNU groups was very similar, leading us to conclude that exercise did not influence this parameter. Although there is a lack of studies corroborating our findings, we suggest that the absence of differences might be attributed to factors such as the timing and duration of exercise, as well as the cancer stage or tumor microenvironment.

Our results indicate that resistance training has a complex impact on the physiological and biochemical parameters in rats with chemically induced mammary cancer. Resistance training has been shown to promote muscle and bone growth, improve certain hematological parameters (such as red and white blood cells, hemoglobin, and platelets), and reduce triglyceride levels [48]. Additionally, resistance training may influence tumor development and progression. These findings highlight the intricate nature of exercise as a preventive approach to cancer, showcasing both its potential benefits and limitations.

To the best of our knowledge, this is the first study to explore the effect of ladder exercise training on an animal model of mammary cancer. While resistance training has been studied extensively in human oncology, its application in veterinary medicine remains largely unexplored. Given that mammary tumors are among the most common cancers in female dogs and cats, understanding how structured exercise impacts tumor biology in companion animal is crucial. Although this study uses a rat model, the findings may have implications for veterinary medicine, particularly in dogs and cats with cancer. Resistance exercise could be a promising adjuvant therapy to improve tumor control and quality of life in animals undergoing cancer treatment, requiring species-specific protocols. Further research is needed to establish safe and effective resistance training protocols tailored to pets, considering factors such as exercise intensity, duration, and individual health conditions. It also should incorporate biomarkers, such as corticosteroids, gene expression, inflammatory cytokines, and angiogenic factors, to elucidate the biological pathways involved. These findings may contribute to the development of non-pharmacological strategies to support cancer prevention and overall well-being in companion animals.

## 5. Conclusions

The findings of this study underscore the complex role of resistance training in the context of chemically induced mammary cancer in rats, providing insights that may be relevant for companion animals. Although resistance training was found to have beneficial effects on muscle strength, blood parameters, and immune function, its impact on tumor development and progression was nuanced. The first tumor appeared only two weeks earlier, and the incidence was slightly higher in the exercised group (EX+MNU) than in the control group (CTR+MNU), the mortality rate was lower in this study, and the malignancy did not prove to be as aggressive. The observed reduction in ERα and PR, coupled with the enhanced presence of CD8 cells, suggests that resistance training may offer protective benefits by boosting anti-tumor immunity. However, the low levels of inflammatory markers like CRP, along with the lack of significant increase in tumor incidence in the exercised group, suggest that there was no inflammatory response, and exercise did not have unintended effects on tumor biology.

These findings emphasize the dualistic nature of resistance training as a potential complementary approach to cancer management. On the one hand, exercise has been demonstrated to promote general health by increasing physical fitness, boosting immunological function, and improving metabolic health. Conversely, the ability of exercise to influence tumor activity, presumably by modifying immune responses or hormone receptor expression, shows that its role in cancer treatment is not easy. Consequently, while resistance training appears to be a promising adjunct therapy for cancer, further study is needed to define its effects and design safe, effective exercise routines that are tailored to cancer the individual needs of cancer patients. In veterinary medicine, where mammary tumors are among the most common cancers in female dogs and cats, understanding the impact of structured exercise could inform new, non-pharmacological strategies for cancer prevention and management. For future work, we suggest increasing the duration and intensity of exercise, studying additional immune markers (e.g., regulatory T cells, M1/M2 macrophages, CD4+ lymphocytes), conducting a molecular profile, and performing a metabolic assessment of the tumors. These insights could contribute to developing safe and effective exercise programs tailored to the needs of pets with cancer, ultimately improving quality of life and clinical outcomes.

## Figures and Tables

**Table 1:** Initial and accurate final body weight and body mass index for all experimental groups (mean ± S.D.).

Parameters	Experimental Groups			
	CTR (n = 7)	CTR+MNU (n = 5)	EX (n = 7)	EX+MNU (n = 6)
Initial body weight (g)	159.90 ± 13.11	148.27 ± 14.31	157.84 ± 18.75	156.08 ± 10.41
Accurate final body weight (g)	278.71 ± 10.47	274.32 ± 31.18	292.86 ± 20.79	260.67 ± 30.76
Body mass index	12.14 ± 1.35	12.84 ± 1.32	13.13 ± 0.75	12.21 ± 1.29

CTR: sedentary control group; CTR+MNU: sedentary induced group; EX: exercised control group and EX+MNU: exercised induced group. Statistically significant differences were not found (*p* > 0.05).

**Table 2:** Organs’ relative weight and bone relative length at the end of the protocol (mean ± S.D.).

Organs	Experimental Groups			
	CTR (n = 7)	CTR+MNU (n = 5)	EX (n = 7)	EX+MNU (n = 6)
Spleen	0.69 ± 0.11	0.68 ± 0.21	0.73 ± 0.11	0.69 ± 0.16
Heart	0.80 ± 0.05	0.78 ± 0.06	0.79 ± 0.09	0.75 ± 0.07
Lungs	1.43 ± 0.13	1.37 ± 0.15	1.46 ± 0.11	1.39 ± 0.09
Liver	7.15 ± 0.91	6.71 ± 0.91	6.30 ± 0.89	6.82 ± 1.19
Soleus	0.21 ± 0.05	0.20 ± 0.02	0.23 ± 0.02	0.20 ± 0.03
Gastrocnemius	3.20 ± 0.20	2.66 ± 0.99	3.56 ± 0.43	3.08 ± 0.24
Biceps brachii	0.28 ± 0.05	0.25 ± 0.06	0.30 ± 0.05	0.26 ± 0.02
Femur	3.41 ± 0.26	3.30 ± 0.07	3.59 ± 0.20	3.32 ± 0.10
Tibia	3.57 ± 0.30 **a**	3.70 ± 0.19	3.96 ± 0.05	3.73 ± 0.15

CTR: sedentary control group; CTR+MNU: sedentary induced group; EX: exercised control group and EX+MNU: exercised induced group. a *p* < 0.05 versus EX group.

**Table 3:** Hemogram analysis (mean ± S.D.).

Parameters	Experimental Groups			
	CTR (n = 7)	CTR+MNU (n = 5)	EX (n = 7)	EX+MNU (n = 6)
Erythrocytes (M/µL)	8.27 ± 0.63	8.16 ± 0.68 a	8.24 ± 0.44 a	9.40 ± 0.38
Hematocrit (%)	44.60 ± 2.61	45.00 ± 4.86 a	44.56 ± 1.86 a	53.10 ± 2.06
Hemoglobin (g/dL)	15.17 ± 0.77	15.20 ± 1.28 a	15.17 ± 0.55 a	17.90 ± 0.60
RDW (%)	20.51 ± 1.51	19.32 ± 2.37	19.59 ± 1.74	20.64 ± 0.44
Reticulocytes (K/µL)	243.53 ± 50.14	267.16 ± 59.83	202.93 ± 32.34	250.90 ± 51.61
Leucocytes (K/µL)	1.86 ± 0.62	2.39 ± 0.66	1.93 ± 1.00	3.27 ± 1.09
Neutrophils (K/µL)	0.41 ± 0.14	0.56 ± 0.20	0.29 ± 0.10 e	0.41 ± 0.09
Lymphocytes (K/µL)	1.34 ± 0.52	1.69 ± 0.52	1.55 ± 0.87	2.76 ± 0.99
Monocytes (K/µL)	0.10 ± 0.05	0.13 ± 0.05	0.09 ± 0.04	0.09 ± 0.03
Eosinophils (K/µL)	0.01 ± 0.00	0.02 ± 0.01	0.01 ± 0.01	0.01 ± 0.00
Basophils (K/µL)	0.01 ± 0.00	0.01 ± 0.00	0.01 ± 0.00	0.01 ± 0.00
NLR	0.34 ± 0.15	0.35 ± 0.12	0.21 ± 0.07	0.16 ± 0.03
Platelets (K/µL)	613.71 ± 83.87	489.40 ± 223.47	600.29 ± 100.12	636.20 ± 53.28
MPV (fL)	8.71 ± 0.21	8.96 ± 0.46	8.61 ± 0.41	8.64 ± 0.31
PDW (fL)	9.86 ± 0.42	10.26 ± 1.00	9.31 ± 0.72	9.84 ± 0.55

CTR: sedentary control group; CTR+MNU: sedentary induced group; EX: exercised control group and EX+MNU: exercised induced group. RDW: red cell distribution width; NLR: neutrophil–lymphocyte ratio; MPV: mean platelet volume; PDW: platelet distribution width. a *p* < 0.05 versus EX+MNU group.

**Table 4:** Serum biochemical parameters analyzed (mean ± S.D.).

Parameters	Experimental Groups			
	CTR (n = 7)	CTR+MNU (n = 5)	EX (n = 7)	EX+MNU (n = 6)
Albumin (g/dL)	4.77 ± 0.68	4.52 ± 0.02	4.10 ± 0.90	3.56 ± 1.05
Cholesterol (mg/dL)	104.77 ± 29.50	90.20 ± 6.64	85.20 ± 26.69	104.47 ± 29.24
CK-MB (U/L)	471.15 ± 160.00	558.94 ± 429.60	230.83 ± 166.20	202.66 ± 145.05
Triglycerides (mg/dL)	77.83 ± 36.38	122.45 ± 61.95	53.54 ± 11.11	88.77 ± 20.62

CTR: sedentary control group; CTR+MNU: sedentary induced group; EX: exercised control group and EX+MNU: exercised induced group. CK-MB: creatinine kinase MB. Statistically significant differences were not found (*p* < 0.05).

**Table 5:** Histological classification of the mammary tumors identified.

Histological Patterns			Experimental Groups			
			CTR (n = 7)	CTR+MNU (n = 5)	EX (n = 7)	EX+MNU (n = 6)
Malignant tumors	In situ carcinoma	Ductal solid and cribriform	0	0	0	1
	Invasive carcinoma	Papillary	0	1	0	2
		Cribriform	0	4	0	3
		Tubular	0	0	0	1
Total malignant tumors:			0	5	0	7

CTR: sedentary control group; CTR+MNU: sedentary induced group; EX: exercised control group and EX+MNU: exercised induced group. Statistically significant differences were not found (*p* > 0.05).

**Table 6:** Evaluation of ERα, PR, and Ki-67 immunoexpression in mammary tumors (mean ± S.D.).

	CTR+MNU (n = 5)		EX+MNU (n = 6)			
	Invasive Carcinoma		In Situ Carcinoma	Invasive Carcinoma		
	Papillary	Cribriform	Ductal solid and Cribriform	Papillary	Cribriform	Tubular
ERα (%)	39.25 ± 11.95	43.27 ± 11.78 ^a^	13.65 ± 7.28	21.13 ± 8.23	20.12 ± 6.77 ^a^	14.95 ± 2.33
PR (%)	60.15 ± 1.20	56.44 ± 9.98 ^b^	31.65 ± 9.12	34.43 ± 17.28	25.48 ± 12.75 ^b^	24.60 ± 7.78
Ki-67 (%)	16.15 ± 1.91 ^c,d^	2.00 ± 1.56 ^c^	1.30 ± 0.42 ^e^	7.15 ± 2.5 ^d,e^	6.22 ± 2.23	2.50 ± 0.14

Values with different letters were considered statistically different (*p* < 0.05). CTR+MNU: sedentary induced group, and EX+MNU: exercised induced group. ERα: estrogen receptor alpha; PR: progesterone receptor.

## Data Availability

All data are contained within this manuscript.

## Abbreviations

The following abbreviations are used in this manuscript:

CBC	Complete blood count
CK-MB	Creatinine kinase-MB
CRP	C-reactive protein
CTR	Sedentary control group
CTR+MNU	Sedentary induced group
ERα	Estrogen receptor alpha
EX	Exercised control
EX+MNU	Exercised induced group
H&E	Hematoxylin and eosin
IL-6	Interleukin 6
MCL	Maximal carrying load
MNU	*N*-methyl-*N*-nitrosourea
MPV	Mean platelet volume
NLR	Neutrophil-lymphocyte ratio
PDW	Platelet distribution width
PR	Progesterone receptor
RDW	Red cells distribution width
VEGF-A	Vascular endothelial growth factor-A
